# *Mycobacterium tuberculosis* Upregulates TNF-α Expression via TLR2/ERK Signaling and Induces MMP-1 and MMP-9 Production in Human Pleural Mesothelial Cells

**DOI:** 10.1371/journal.pone.0137979

**Published:** 2015-09-14

**Authors:** Wei-Lin Chen, Joen-Rong Sheu, Ray-Jade Chen, Shih-Hsin Hsiao, Che-Jen Hsiao, Yung-Chen Chou, Chi-Li Chung, George Hsiao

**Affiliations:** 1 Graduate Institute of Medical Sciences and Department of Pharmacology, College of Medicine, Taipei Medical University, Taipei, Taiwan; 2 Department of Surgery, School of Medicine, College of Medicine, Taipei Medical University, Taipei, Taiwan; 3 Division of General Surgery, Department of Surgery, Taipei Medical University Hospital, Taipei, Taiwan; 4 Division of Pulmonary Medicine, Department of Internal Medicine, Taipei Medical University Hospital, Taipei, Taiwan; 5 School of Respiratory Therapy, College of Medicine, Taipei Medical University, Taipei, Taiwan; Institut de Pharmacologie et de Biologie Structurale, FRANCE

## Abstract

**Background:**

Tumor necrosis factor (TNF)-α and matrix metalloproteinases (MMPs) are elevated in pleural fluids of tuberculous pleuritis (TBP) where pleural mesothelial cells (PMCs) conduct the first-line defense against *Mycobacterium tuberculosis* (MTB). However, the clinical implication of TNF-α and MMPs in TBP and the response of PMCs to MTB infection remain unclear.

**Methods:**

We measured pleural fluid levels of TNF-α and MMPs in patients with TBP (n = 18) or heart failure (n = 18) as controls. Radiological scores for initial effusion amount and residual pleural fibrosis at 6-month follow-up were assessed. In vitro human PMC experiments were performed to assess the effect of heat-killed *M*. *tuberculosis* H37Ra (MTBRa) on the expression of TNF-α and MMPs.

**Results:**

As compared with controls, the effusion levels of TNF-α, MMP-1 and MMP-9 were significantly higher and correlated positively with initial effusion amount in patients with TBP, while TNF-α and MMP-1, but not MMP-9, were positively associated with residual pleural fibrosis of TBP. Moreover, effusion levels of TNF-α had positive correlation with those of MMP-1 and MMP-9 in TBP. In cultured PMCs, MTBRa enhanced TLR2 and TLR4 expression, activated ERK signaling, and upregulated TNF-α mRNA and protein expression. Furthermore, knockdown of TLR2, but not TLR4, significantly inhibited ERK phosphorylation and TNF-α expression. Additionally, both MTBRa and TNF-α markedly induced MMP-1 and MMP-9 synthesis in human PMCs, and TNF-α neutralization substantially reduced the production of MMP-1, but not MMP-9, in response to MTBRa stimulation.

**Conclusion:**

MTBRa activates TLR2/ERK signalings to induce TNF-α and elicit MMP-1 and MMP-9 in human PMCs, which are associated with effusion volume and pleural fibrosis and may contribute to pathogenesis of TBP. Further investigation of manipulation of TNF-α and MMP expression in pleural mesothelium may provide new insights into the mechanisms and rational treatment strategies for TBP.

## Introduction

Tuberculosis (TB) remains a major global public health issue and continues to cause significant morbidity and mortality worldwide [1]. It has been well documented that tumor necrosis factor (TNF)-α and matrix metalloproteinases (MMPs) are essential in the pathogenesis of tuberculosis [2, 3]. Tuberculous infection commences when activated macrophages engulf *Mycobacterium tuberculosis* (MTB) and produce TNF-α and MMP-9 to recruit more macrophages, other immune cells and stromal cells such as fibroblasts, astrocytes or epithelial cells, to aggregate around the infected cells and form granulomas [4]. Within the granuloma, the cell-cell interactions develop to amplify the immune responses and enhance TNF-α, MMP-1 and MMP-9 expression in immune cells and stromal cells [3]. During reactivation, granulomas break down and extracellular matrix (ECM) were degraded by highly upregulated MMP-1, leading to MTB transmission, tissue remodeling and subsequent fibrosis [5].

Tuberculous pleuritis (TBP) is the most common form of extrapulmonary tuberculosis and a usual cause of pleural fibrosis [6]. The pleura space is lined by a metabolically active monolayer of pleural mesothelial cells (PMCs), which may serve as the first-line defense against invading microorganisms [7]. In response to pleural infection, PMCs not only provide physical barriers but also produce cytokines, chemokines, ECM and proteases that participate in the induction and resolution of inflammation and tissue repair [8]. The TNF-α level in the pleural fluid from patients with TBP are significantly higher than in those with pleural malignancy [9]. Moreover, patients with TBP who go on to develop pleural fibrosis have substantially higher TNF-α level in the pleural fluid than those who do not [9]. On the other hand, MMP-1 and MMP-9 are elevated in TBP as compared with transudative effusions [10], and MMP-9 is prominently expressed in the granulomas of tuberculous pleural tissues [11]. All these findings suggest that PMCs and its relationship with TNF-α and MMPs may contribute to the pathogenesis of TBP.

There have been studies on the expression of TNF-α and MMPs in monocytes and fibroblasts composing the pulmonary tuberculous granuloma [12, 13]. However, the role of PMCs in TBP and the regulation of TNF-α expression and MMP elaboration in PMCs upon MTB infection have never been investigated. We hypothesized that TNF-α can be induced by MTB in PMCs and may drive PMCs to elicit MMPs expression and secretion. Therefore, we explored the effect of heat-killed *M*. *tuberculosis* H37Ra on TNF-α expression and subsequent production of MMPs in human PMCs, the underlying mechanisms, and the clinical implications in TBP.

## Materials and Methods

### Patient Recruitment

Consecutive patients with pleural effusions of unknown causes admitted to Taipei Medical University Hospital who underwent thoracentesis and/or closed pleural biopsy were eligible for this study. TBP was diagnosed as previously described [14]. Patients with congestive heart failure (CHF) and a transudative pleural effusion were enrolled as controls [10]. Ethics approval (CRC-05-11-01) was obtained from the Institutional Review Board of Taipei Medical University (Taipei, Taiwan), and all patients gave written informed consent before entering the study.

### Chest Radiograph Scoring

The posteroanterior chest radiographs (CXR) were performed at admission, on discharge and 6 months later, respectively, and were scored by two radiologists blind to any clinical information to determine (a) the largest linear width of pleural opacity and (b) pleural CXR score: the estimated overall percentage of pleural shadowing in the hemithorax. Residual pleural thickening (RPT) was defined as a lateral pleural thickening of ≥ 10 mm shown on CXR at the end of 6-month follow-up [15].

### Reagents

Heat-killed *M*. *tubuculosis* H37Ra (MTBRa) obtained from Difco Lab (Detroit, Mich) was dissolved in phosphate-buffered saline (PBS) and used as a stimulant [16]. Recombinant human TNF-α were purchased from Pepro Tech EC (London, UK), respectively. Except for ERK (Cell Signaling Technology, Beverly, MA) and HRP conjugated anti-mouse and anti-rabbit IgG (Jackson Immunoresearch, USA), other antibodies were purchased from GeneTex (GeneTex Inc). SB203580, SP600125, PD98059, LY294002 and parthenolide were obtained from Calbiochem (San Diego, CA).

### Human Pleural Mesothelial Cells (PMCs)

The cell culture of Met-5A human PMC line (#CRL-9444TM) was obtained from American Type Culture Collection (ATCC, Manassas, VA) and primary human PMCs were collected from the pleural fluids of patients with congestive heart failure. The ethics approval (IRB No.: CRC-05-11-01) for the collection and generation of the primary-cultured human PMCs was obtained from the Institutional Review Board of Taipei Medical University (Taipei, Taiwan), and the written informed consent was acquired from all patients recruited. The human pleural fluids were centrifuged and cells were grown in medium 199 containing 10% FBS at 37°C in the humidified incubator of 5% CO_2_. Mesothelial cells were used at passages three to six and were characterized by the cobblestone morphology, the presence of cytokeratin, and the absence of factor VIII, as described in our previous study [17].

### Enzyme-linked Immunosorbent Assay (ELISA)

Pleural fluid samples from patients with TBP and CHF were collected as described in our previous report [18]. The commercially available ELISA kits were used to measure effusion levels of TNF-α, MMP-1 MMP-7, and MMP-9 (R & D System; Minneapolis, MN, USA) [18]. The levels of TNF-α in the supernatant of human PMCs after MTBRa stimulation were analyzed with ELISA methods as well.

### Western Blot

The proteins were separated in denaturing sodium dodecyl sulfate (SDS) polyacrylamide gels and electrophoretically transferred onto nitrocellulose membranes. Blotting membranes were incubated with specific primary and HRP-conjugated secondary antibodies. Bound antibody was visualized by chemiluminescence and exposure of the membrane to light-sensitive film. The quantitative densitometric analysis was performed as previously described [19].

### Reverse Transcription-Polymerase Chain Reaction (RT-PCR)

Total RNA was isolated from Met-5A cells using the TRIsure^®^reagent (Bioline, London, UK) and RNA (1 μg) was used for cDNA synthesis (Super Script On-Step RT-PCR system, GIBCOTM). The following primers were used: for TNF-α, sense 5′-AGCCCATGTT GTAGCAAACC-3′ and antisense 5′-CCAAAGTAGACCTGCCCAGA-3′; and for GAPDH, sense 5′-GCCGCCTGGTCACCAGGGCTG-3′ and antisense 5′-ATGGACTGTGGTCATGAGCCC-3′. The PCR was performed with the following conditions: 30 cycles of a 15-s denaturation step at 95°C, a 60-s annealing step at 48°C, and a 60-s extension step at 72°C to amplify TNF-α cDNA, followed by 25 cycles of a 15-s denaturation step at 95°C, a 30-s annealing step at 60°C, and a 60-s extension step at 72°C to amplify GAPDH cDNA. The quantitative analyses were performed as previously described [20].

### Flow Cytometry

Met-5A cells were collected and washed with 2% FBS flow cytometry wash buffer. The resulting cells were sequentially stained with primary antibodies against control IgG, TLR2 and TLR4 for 30 min at room temperature, respectively. After additional washing steps, cells were incubated with the secondary antibody, FITC-conjugated goat anti-rabbits IgG, for another 30 min. The cells were washed and 10,000 events were analyzed by flow cytometry using FACSCalibur flow cytometer (Becton Dickinson).

### RNA Interference

MeT-5A cells were transfected with a final concentration of 25 nmol of small interfering RNA (siRNA) against TLR2 or TLR4, or scrambled siRNA using the DharmaFECT^®^ siRNA transfection reagent (Thermo Scientific, USA) according to the manufacturer’s instructions. After 48 h incubation with the transfection reagent, cells were stimulated with MTBRa for different times. Specific gene silencing was confirmed by Western blotting.

### Statistical Analyses

Data analyses were performed with SigmaStat 3.5 (SYSTAT Software, San Jose, CA). Data were expressed as mean ± SD, median or frequency (%), as indicated. Comparisons of continuous data were made using an unpaired *t* test or Mann–Whitney U test between two groups, where appropriate, and one-way analysis of variance among three groups. Categorical variables between two groups were examined using χ^2^ method. Correlations were analyzed with the Spearman rank correlation coefficients. p < 0.05 was considered statistically significant.

## Results

### Patient Characteristics

Clinical data and pleural fluid characteristics of 18 patients with TBP and 18 control patients with CHF were shown in Table 1. No statistical differences among the two groups were found in terms of age, gender, duration of illness before management. The initial amount of effusion, measured as area of effusion shadowing on CXR, the effusion pH value, and the effusion glucose level were significantly lower in TBP than in CHF-related effusions. In contrast, as compared with the effusion of CHF patients, the leukocyte count, the levels of lactate dehydrogenase (LDH) and ADA were significantly higher in TBP. After 6-months anti-TB medications, 6 of the 18 patients with TBP developed significant RPT (≥10 mm).

**Table 1:** Clinical Data and Pleural Fluid characteristics*. *Definition of abbreviations*: TBP, tuberculous pleuritis; CHF, congestive heart failure; LDH, lactate dehydrogenase; ADA, adenosine deaminase; RPT, residual pleural thickening.

Characteristics	TBP	CHF	p Value
**Subjects, n**	18	18	
**Mean age, years**	62 ± 24	72 ± 20	0.197
**Male, n (%)**	15 (83)	10 (56)	0.073
**Symptom onset to management, days**	12 ± 8	10 ± 4	0.130
**Area of effusion shadowing,** ^†^ **%**	47.8 ± 17.6	62.4 ± 20.4	0.038
**Pleural effusion**			
pH value	7.33 ± 0.12	7.38 ± 0.04	0.019
Glucose, mg/dL	109 ± 44	169 ± 82	0.023
LDH, IU/dL	467 ± 160	141 ± 84	0.003
Leukocyte count, cells/μL	2304 ± 870	300 ± 53	0.028
ADA, IU/L	140 ± 60	25 ± 8	< 0.001
**Area of pleural thickening at 6 months,** ^†^ **%**	4.5 ± 2.2	-	
**RPT ≥ 10 mm, n (%)**	6 (33)	-	

* Values are presented as mean ± SD unless otherwise specified.

^†^ on posteroanterior chest radiography.

### Pleural Fluid Levels of TNF-α and MMPs in TBP and Relationship with Radiological Scores

**Fig 1:**
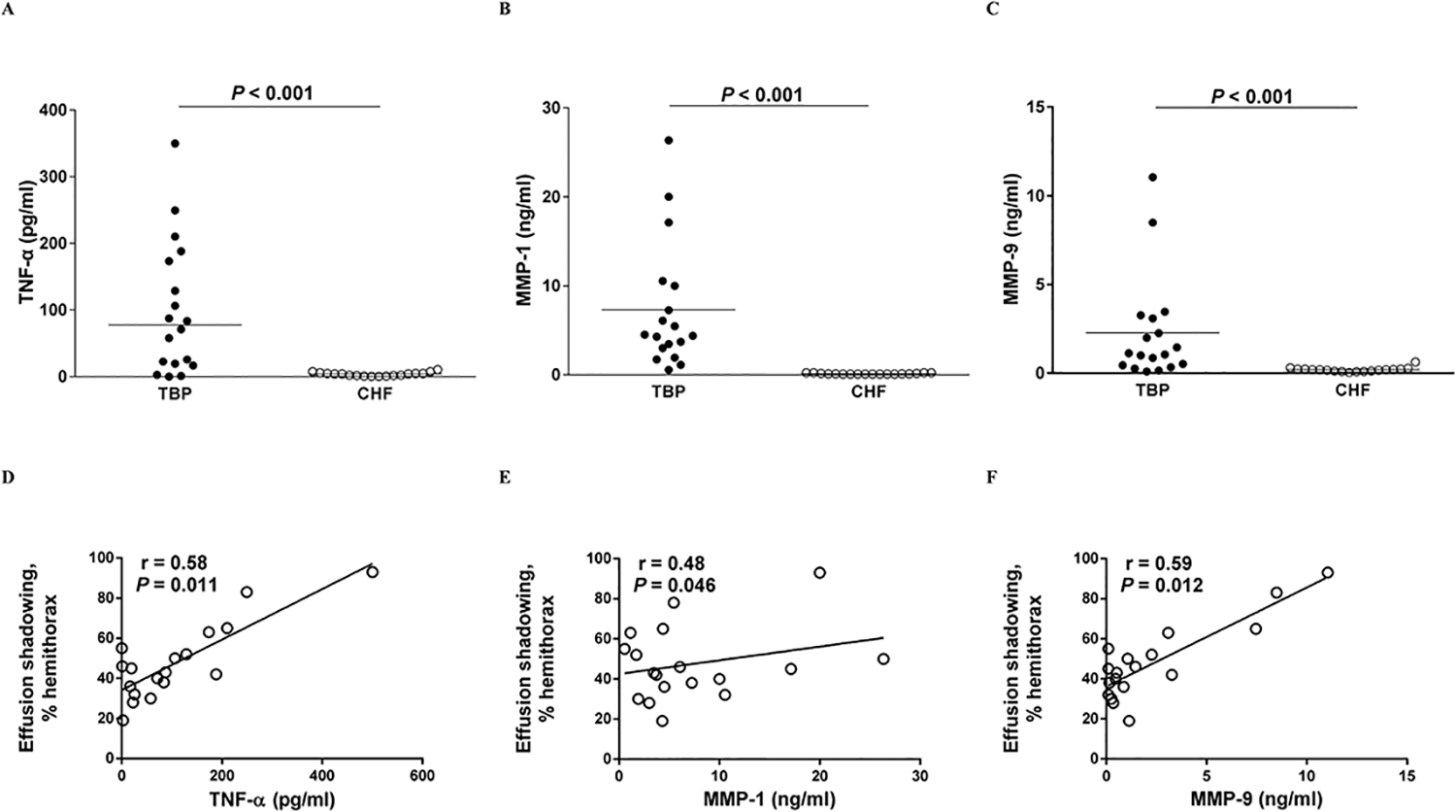
Effusion levels of TNF-α, MMPs and their relationship with effusion radiological scores in TBP patients. Levels of (A), TNF-α, (B), MMP-1 and (C), MMP-9 in pleural effusion of patients with TBP (n = 18) or CHF (n = 18), and the correlation between effusion levels of (D), TNF-α, (E), MMP-1 or (F), MMP-9 and effusion shadowing on chest radiograph in patients with TBP. TNF, tumor necrosis factor; MMP, matrix metalloproteinase; TBP, tuberculous pleuritis; CHF, congestive heart failure.

As shown in Fig 1A, 1B and 1C, the median levels of TNF-α, MMP-1, MMP-9, in pleural fluids from patients with TBP were significantly higher than in those with CHF (TNF-α: 77.4 vs. 4.2 pg/ml, p<0.001; MMP-1: 4.4 vs. 0.1 ng/ml, p<0.001; MMP-9: 1.1 vs. 0.2 ng/ml, p<0.001), respectively. By contrast, the effusion levels of MMP-7 were comparable between the two groups.

**Fig 2:**
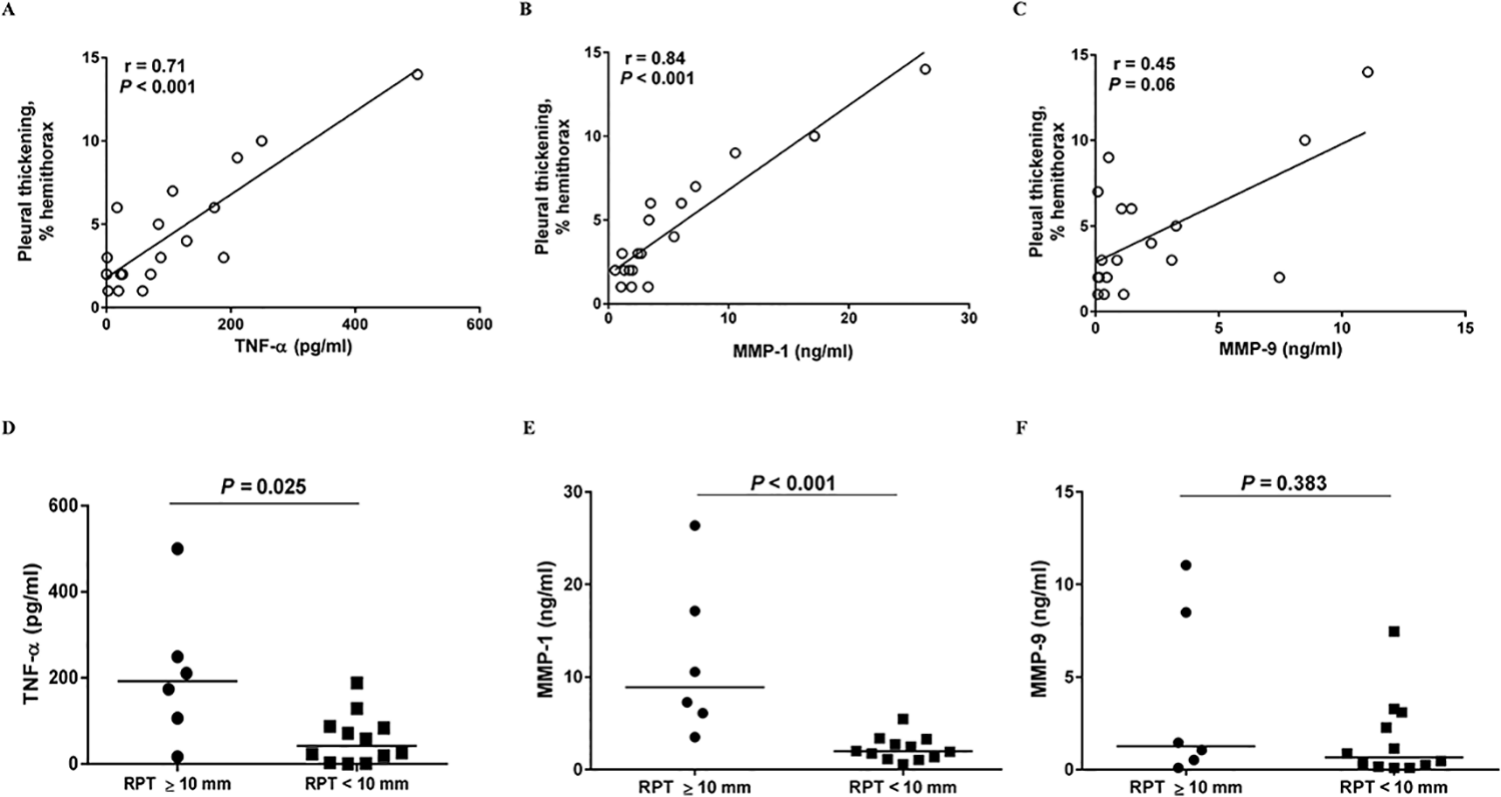
Relationship between effusion levels of TNF-α and MMPs and residual pleural thickening in TBP patients. Correlation between pleural fluid levels of (A), TNF-α, (B), MMP-1 or (C), MMP-9 and pleural thickening on chest radiograph in patients with TBP (n = 18), and pleural fluid levels of (D), TNF-α, (E), MMP-1 and (F), MMP-9 between TBP patients with RPT ≥10 mm (n = 6) and those with RPT <10 mm (n = 12). TNF, tumor necrosis factor; MMP, matrix metalloproteinase; TBP, tuberculous pleuritis; RPT, residual pleural thickening.

In TBP, the pleural fluid levels of TNF-α, MMP-1 and MMP-9 all correlated positively with the effusion shadowing on CXR, though MMP-1 had a weak correlation (Fig 1D, 1E and 1F). Additionally, the levels of TNF-α and MMP-1, but not MMP-9, correlated positively with the pleural thickening on CXR after 6-months anti-TB medications (Fig 2A, 2B and 2C). Moreover, the effusion levels of TNF-α and MMP-1 were significantly higher in TBP patients with RPT ≥ 10 mm than those with RPT <10 mm, whereas the MMP-9 values were comparable between two groups (Fig 2D, 2E and 2F). These findings may indicate that all these effusion mediators, especially TNF-α, are essential in the pathogenesis of TBP, in which their cross relationship with PMCs may play an important role.

### MTBRa Upregulates TNF-α Production in Human PMCs

**Fig 3:**
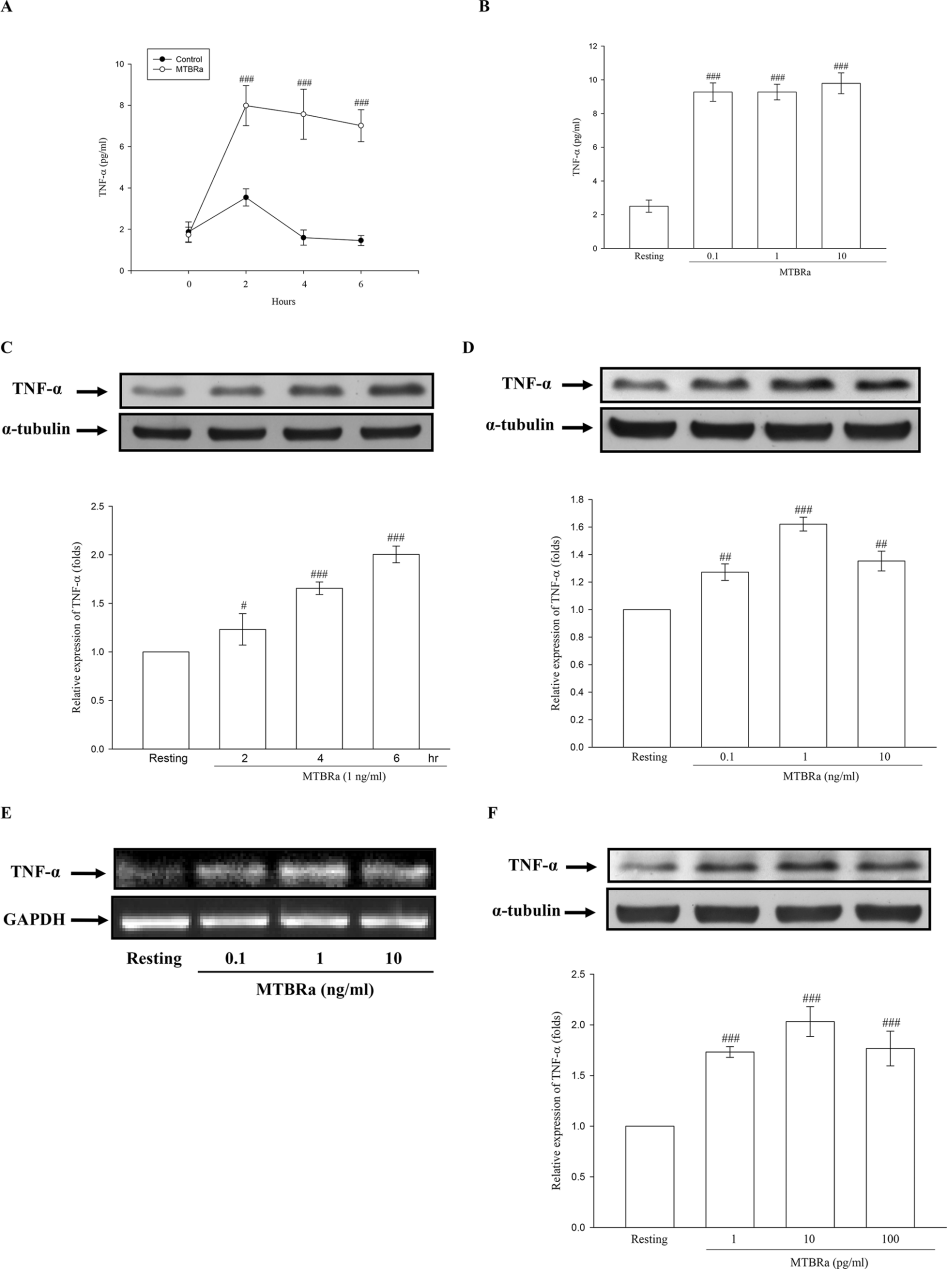
Expression and release of TNF-α in MTBRa-Stimulated Human PMCs. Levels of TNF-α in the culture supernatants of MeT-5A cells stimulated by (A), MTBRa (1 ng/ml) for the indicated times or (B), MTBRa with various concentrations (0.1–10 ng/ml) for 6 h. TNF-α protein expression in MeT-5A cells treated with (C), MTBRa (1 ng/ml) for the indicated times or (D), MTBRa (0.1–10 ng/ml) for 6 h. (E), TNF-α mRNA expression in MeT-5A cells treated with MTBRa (0.1–10 ng/ml) for 4 h. (F), TNF-α protein expression in primary-cultured human PMCs stimulated with MTBRa (0.1–10 ng/ml) for 6 h. Supernatant TNF-α level was analyzed by ELISA and cell lysate TNF-α protein level was assessed by Western blot. TNF-α mRNA concentrations were analyzed by semiquantitative reverse transcriptase PCR and normalized with GAPDH mRNA. Data are shown as mean ± SD of three to five independent experiments. ^#^p<0.05 and ^###^p<0.001 compared with the control or resting group. PMC, pleural mesothelial cell; MTBRa, heat-killed *M*. *tuberculosis* H37Ra; TNF, tumor necrosis factor; GAPDH, glyceraldehyde 3-phosphate dehydrogenase.

Furthermore, we explored the production of TNF-α in human PMCs upon exposure to MTBRa. As shown in Fig 3A, the addition of MTBRa (1 ng/ml) for 2 to 6 hours markedly stimulated TNF-α production by Met-5A cells. Besides, the TNF-α level in the supernatants increased substantially when Met-5A cells were stimulated with different concentrations of MTBRa for 6 h as compared with the resting condition (Fig 3B). In Fig 3C, MTBRa (1 ng/ml) significantly induced TNF-α protein expression in MeT-5A cells in a time-dependent manner. Moreover, TNF-α synthesis increased substantially after stimulation with different concentrations of MTBRa (0.1, 1, and 10 ng/ml) for 6 h (Fig 3D). Furthermore, MTBRa significantly increased the expression of TNF-α mRNA in MeT-5A cells, as compared with the resting condition (Fig 3E). Consistently, MTBRa concentration (1, 10, and 100 pg/ml)-dependently enhanced the TNF-α production in the primary-cultured human PMCs (Fig 3F). These findings suggest that, upon *M*. *tuberculosis* infection, human PMCs may substantially contribute to the increased production of TNF-α in TBP. Since the number of times primary human PMCs can be passage is limited and the cellular responses of MeT-5A cells were in parallel with those of primary PMCs, that has been validated in several previous studies [18, 19, 21], the following experiments on cellular signaling mediating MTBRa action were performed by using MeT-5A cells as a model of human pleural biology.

### Signal pathways mediating TNF-α expression in MTBRa-stimulated human PMCs

**Fig 4:**
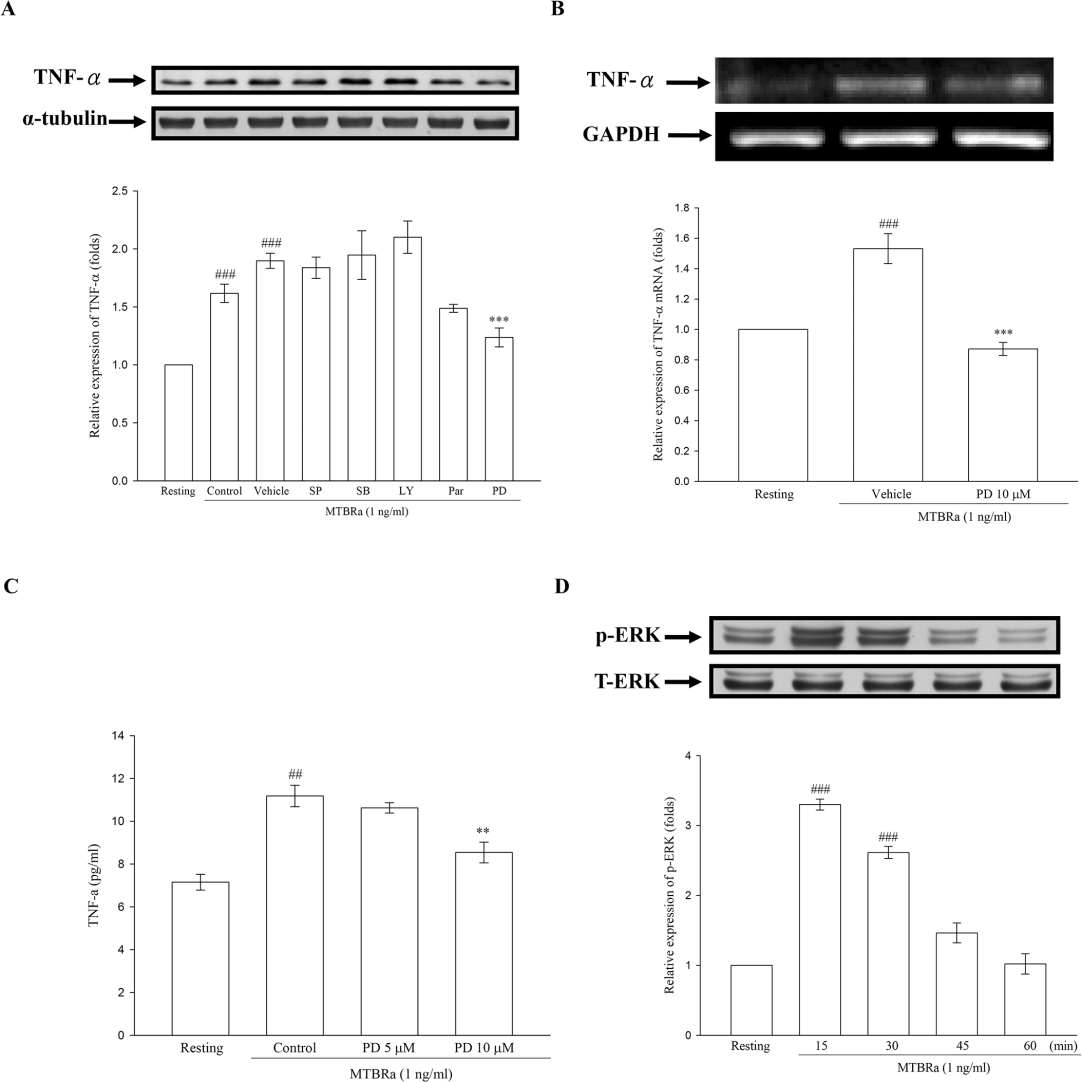
Signal pathways mediating TNF-α expression in MTBRa-stimulated human PMCs. (A), MeT-5A cells were pretreated with vehicle, SP600125 (SP), SB20358 (SB), LY294002 (LY), Parthenolide (Par), PD98059 (PD), respectively, then stimulated with MTBRa (1 ng/ml) for 24 h. TNF-α protein expression was assessed by Western blot. (B), MeT-5A cells were pretreated with PD98059 (PD) (10 μM), followed by stimulation with MTBRa (1 ng/ml) for 4h. TNF-α mRNA concentrations were analyzed by RT-PCR. (C), MeT-5A cells were pretreated with PD98059 (PD) (5 and 10 μM) following stimulation with MTBRa (1 ng/ml) for 6 h, supernatant TNF-α level was analyzed by ELISA. (D), MeT-5A cells were treated with MTBRa (1 ng/ml) for the indicated times. ERK expression was analyzed by Western blotting with antibodies specific for phosphorylated or total proteins. ^##^p <0.01, ^###^p<0.001 compared with the resting group; **p<0.001, ***p<0.001 compared with the vehicle (DMSO) group. PMC, pleural mesothelial cell; TLR, toll-like receptor; MTBRa, heat-killed *M*. *tuberculosis* H37Ra; TNF, tumor necrosis factor; ERK, extracellular-signal-regulated kinase.

To clarify the signalings mediating TNF-α expression in MTBRa-stimulated human PMCs, we examined the several signaling pathways, including NF-κB, PI3K/AKT or MAPKs, by using their specific pharmacologic inhibitors. As shown in Fig 4A and 4B, both the protein and mRNA expression of TNF-α induced by MTBRa were markedly attenuated by pretreatment with MEK inhibitor (PD98059, 10 μM). Similar inhibitory effect by MEK inhibitor on MTBRa–induced TNF-α secretion was shown in Fig 4C. However, other inhibitors did not affect MTBRa–induced TNF-α protein synthesis. Consistently, MTBRa significantly induced phosphorylation of ERK 1/2 within 30 min, compared with the resting condition (Fig 4D). These results demonstrated MTBRa induced TNF-α expression via ERK-dependent pathway in human PMCs.

### MTBRa Activates TLR2/ERK Signaling to Induce TNF-α Expression

**Fig 5:**
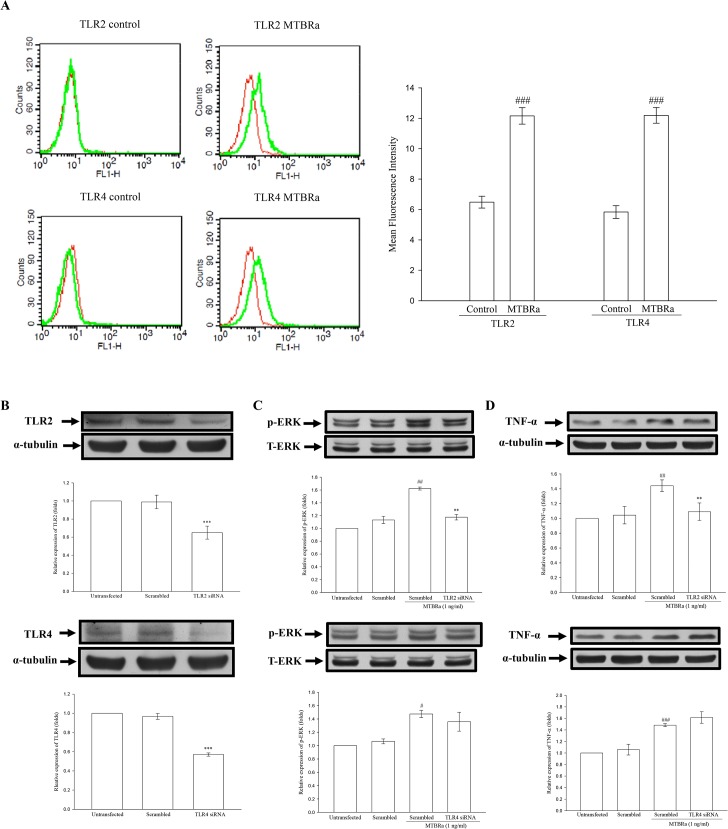
TLR/ERK Signaling Activating TNF-α Expression in MTBRa-Stimulated Human PMCs. (A), Flow cytometric analyses of expression of TLR2 or TLR4 (green) on cell surface of MeT-5A cells treated with MTBRa (1 ng/ml) for 3 h or left untreated (control). Isotype control IgG (red). Quantitative analysis of TLR2 or TLR4 expression was expressed as percentage of the total cells. A representative of three experiments is depicted. ^###^p<0.001 compared with the control group. MeT-5A cells were transfected with scrambled siRNA, TLR2 siRNA (25 nM) or TLR4 siRNA (25 nM). After 30 min, 3 and 6 h of stimulation with MTBRa, the cellular extracts were prepared and the protein amounts of (B), TLR2 and TLR4, (C) ERK, and (D), TNF-α were determined by Western blotting. The data represent four independent experiments. ^##^p <0.01, ^###^p<0.001 compared with the control (scrambled RNA) group; **p<0.01, ***p<0.001 compared with scrambled RNA-transfected MTBRa-treated group. PMC, pleural mesothelial cell; TLR, toll-like receptor; MTBRa, heat-killed *M*. *tuberculosis* H37Ra; ERK, extracellular-signal-regulated kinase; TNF, tumor necrosis factor.

*M*. *tuberculosis* is known to induce proinflammatory cytokines expression through cell surface receptors, especially Toll-like receptors (TLRs) [22–23]. Firstly, we examined the basal and MTBRa-induced expression of TLRs in MeT-5A. The flow cytometric analyses revealed that, compared with controls, MTBRa significantly upregulated the expression of both TLR2 and TLR4 on MeT-5A cell surface (Fig 5A).

Furthermore, we used specific siRNA for TLR2 or TLR4 to confirm whether and which TLRs are involved in MTBRa-induced TNF-α expression in human PMCs. The knockdown efficiencies of siRNAs were demonstrated as the marked reduction of TLR2/4 protein levels in human PMCs transfected with TLR2/4 siRNA, as compared with those transfected with a non-targeting scrambled siRNA (Fig 5B). Pretreatment with TLR2 siRNA significantly decreased ERK phosphorylation and TNF-α expression at 30 min and 6 hours after MTBRa administration, respectively (Fig 5C and 5D, upper panel). In contrast, although TLR4 expression increased significantly upon MTBRa stimulation (Fig 5A), knockdown of TLR4 did not abrogate MTBRa-induced ERK phosphorylation and TNF-α production in MeT-5A cells (Fig 5C and 5D, lower panel). These findings suggest that TLR2, but not TLR4, is the main receptor for MTBRa to mediate ERK activation and TNF-α expression in human PMCs.

### Relationship between TNF-α and MMP-1/-9 in TBP

**Fig 6:**
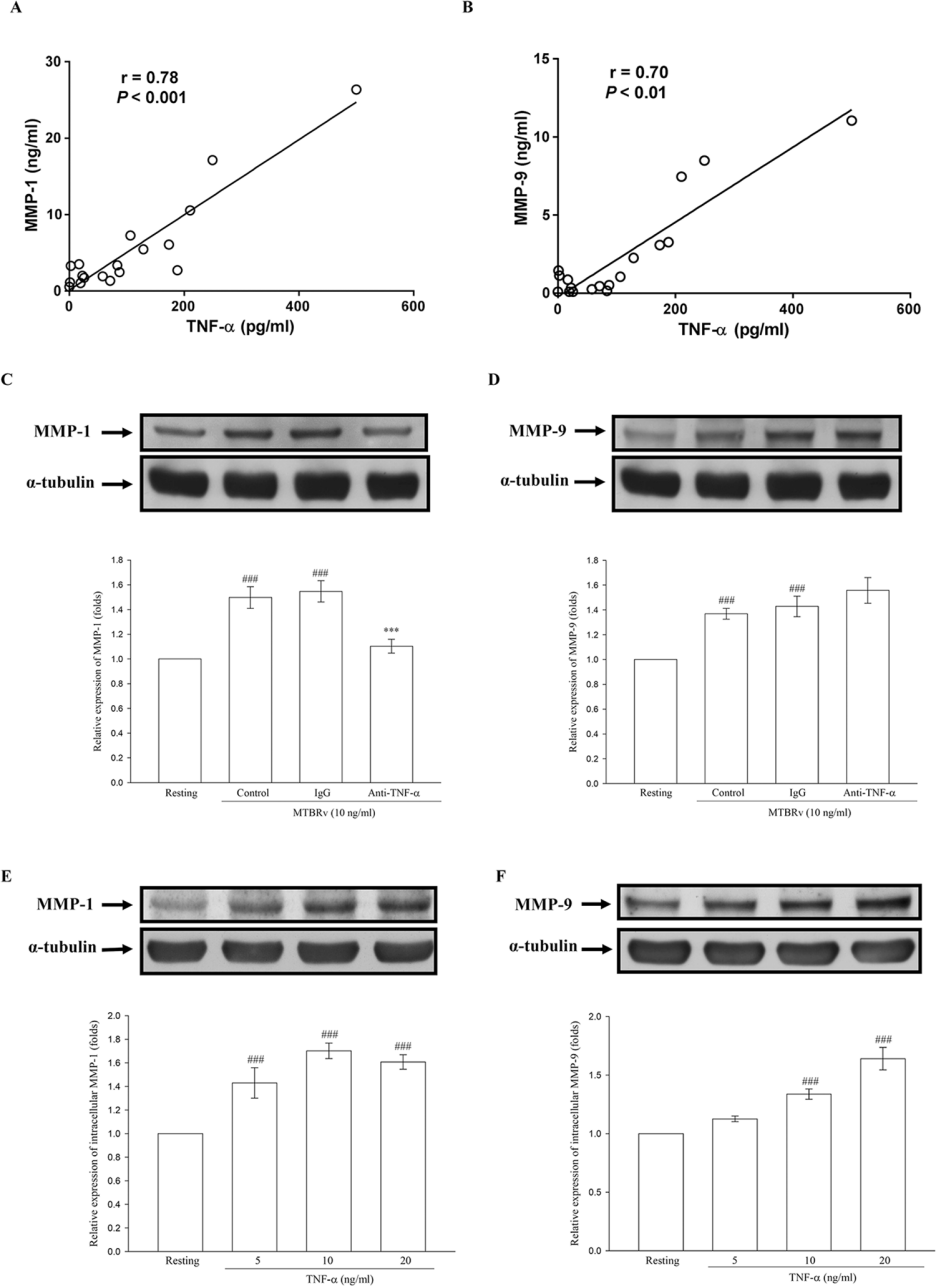
Relationship between pleural fluid levels of TNF-α and MMP-1/-9 in TBP. Correlation between TNF-α and (A), MMP-1 and (B), MMP-9 in TBP (n = 18). Effects of Heat-killed M. tuberculosis H37Ra or TNF-α on the expression of MMP-1 and MMP-9 in human pleural mesothelial cells. (C) and (D), MeT-5A cells were pretreated with vehicle (control), control IgG (12 μg/ml) or anti-TNF-α Ab (12 μg/ml) for 30 min, then stimulated with MTBRa for 24 hours. (E) and (F), MeT-5A cells were stimulated with TNF-α (5–20 ng/ml) for 24 h. The level of MMP-1 and MMP-9 was assessed by Western blot. Data are shown as mean ± SD of three to four independent experiments. ^###^p<0.001 compared with the resting group; ***p<0.001 compared with the MTBRa-treated group (control). TNF, tumor necrosis factor; MMP, matrix metalloproteinase; TBP, tuberculous pleuritis; MTBRa, heat-killed *M*. *tuberculosis* H37Ra.

Since MTBRa could stimulate human PMCs to elaborate TNF-α, we further explored the relationship between TNF-α and MMPs in TBP. The levels of TNF-α were positively correlated with those of MMP-1 and MMP-9 (r = 0.78, p<0.001 and r = 0.70, p<0.01), respectively (Fig 6A and 6B). To determine whether this significant correlation was a causal or associational finding in TBP, further tests of the effect of MTBRa or TNF-α on the expression of MMP-1 and -9 in PMCs were mandatory.

Accordingly, we investigated whether MTBRa or TNF-α can induce MMP-1 and MMP-9 expression in PMCs. As shown in Fig 6C and 6D, MTBRa (10 ng/ml) significantly increased MMP-1 and MMP-9 expression in PMCs as compared with the resting condition. By contrast, pretreatment with anti-TNF-α Ab (12.5 μg/ml) significantly reduced MTBRa-induced MMP-1 expression. However, MMP-9 production is not suppressed by neutralization of TNF-α. Furthermore, administration of recombinant human TNF-α alone substantially increased MMP-1 and MMP-9 production in PMCs in a concentration-dependent manner (Fig 6E and 6F). Collectively, these data suggest that TNF-α is a potent stimulator of MMP-1 and MMP-9 production by PMCs, and that the mycobacterial induction of MMPs in TBP, especially MMP-1, may be mediated by PMC elaboration of TNF-α.

## Discussion

The present clinical data demonstrated that effusion levels of TNF-α, MMP-1 and MMP-9 were significantly elevated in TBP. TNF-α, MMP-1 and MMP-9 were associated with pleural fluid volume, while TNF-α and MMP-1, but not MMP-9, correlated with area of pleural fibrosis. Moreover, substantial positive correlation existed between TNF-α and MMP-1/-9 in TBP. The *in vitro* data showed that MTBRa upregulated TNF-α expression in human PMCs through activation of TLR2/ERK signaling. Both MTBRa and TNF-α significantly induced the synthesis of MMP-1 and MMP-9, and neutralization of TNF-α substantially reduced MMP-1 production elicited by MTBRa. To the best of our knowledge, this is the first study to show that MTB could stimulate human PMCs to elaborate TNF-α and drives the expression of MMP-1 and MMP-9, which indicates the potential role of PMCs in the pathogenesis of TBP.

The clinical data in the present study demonstrate the elevated effusion levels of TNF-α, MMP-1 and MMP-9 in TBP and the association of these mediators with amount of pleural effusion and area of pleural fibrosis. These results highlighted the role of TNF-α, MMP-1 and MMP-9 in the pathogenesis of TBP. TNF-α is the key cytokine for early inflammatory responses during MTB infection [2]. However, in TBP, the production of TNF-α and the activated signalings in PMCs upon exposure to MTB has never been investigated. Our in vitro data revealed that MTBRa time- and concentration-dependently induced TNF-α production in human PMCs, implying that PMCs may initiate the immune response against MTB infection in the pleural space. Consequently, the mechanism by which PMCs identify MTB as a pathogen needs to be explored.

Previous studies have been demonstrated that TLR2 is the principal receptor for activation of macrophage or alveolar epithelial cells by MTB infection [24–25]. Moreover, MAPKs such as p38 and ERK are signaling mediators that play key roles in the elaboration of proinflammatory cytokines such as TNF-α and IL-10 [26]. In line with the earlier report [27], our data showed that the expression of both TLR2 and TLR4 on PMCs were substantially enhanced upon MTBRa stimulation, and that MTBRa further activated ERK signaling to elicit TNF-α production. However, only knockdown of TLR2, but not TLR4, could significantly reduce MTBRa-induced ERK phosphorylation and TNF-α expression, suggesting that MTBRa upregulates TNF-α expression from human PMCs via TLR2-ERK-dependent signaling pathway. To our knowledge, this is the first study to demonstrate the participation of TLR signalings in the inflammatory responses of human PMCs to MTB infection.

MMP-9 is abundantly increased and associated with granuloma formation in TBP [28], signifying its role in early inflammatory responses to MTB infection of the pleura. In keeping with the earlier report [29], our data showed that MMP-9 was elevated, and moreover, correlated substantially with the pleural fluid volume of TBP. This may be ascribed to the implication of MMP-9 in the proteolytic processes during granuloma formation, which may degrade basement membranes of endothelial cells or PMCs and facilitate fluid entry into the pleural space. On the other hand, MMP-1 is the main protease for tissue destruction and remodeling in tuberculosis [5]. Moreover, it was reported that patients with MMP-1 (-1607G) gene polymorphism are prone to develop lung fibrosis after pulmonary MTB infection [30]. In accordance with the previous studies [29–31], our results showed that the MMP-1 level in TBP was significantly associated with the degree of residual pleural fibrosis and was substantially higher in patients with RPT ≥ 10 mm than those without. In contrast, MMP-9 level did not relate to pleural fibrosis, suggesting the differential roles of MMP-1 and MMP-9 in the pathological processes of TBP. Collectively, these findings indicate that MMP-1 and MMP-9 are closely associated with clinical and radiological markers, are implicated in MTB-driven pleura matrix destruction, and may be key factors in the pathogenesis of TBP.

**Fig 7:**
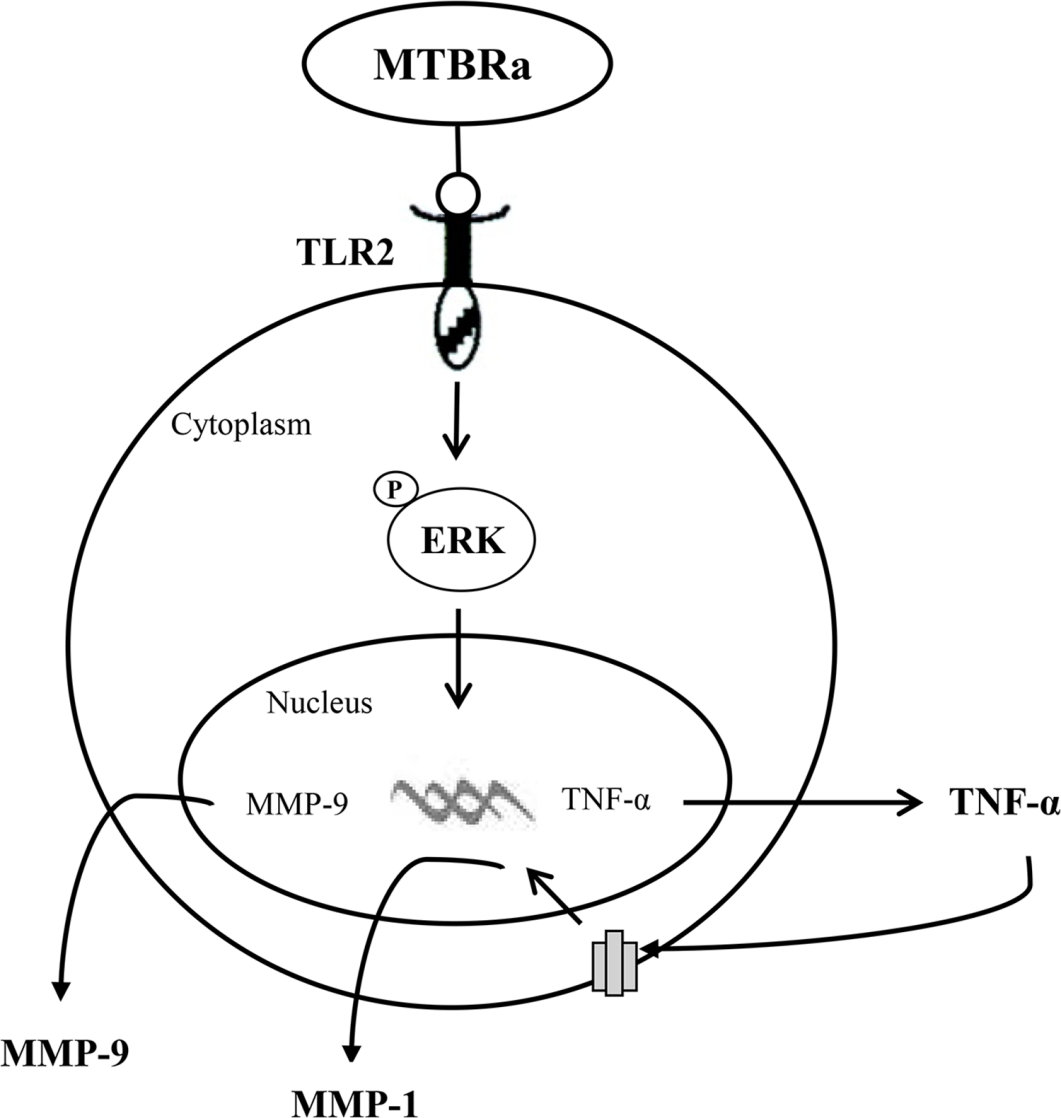
Schematic diagram of the activated signalings in human pleural mesothelial cells upon *Mycobacterium tuberculosis* infection. MTBRa induces TNF-α expression in human pleural mesothelial cells through activation of TLR2/ERK signaling pathway, and increases MMP-1 and MMP-9 production (see text for further explanation). MTBRa, heat-killed *M*. *tuberculosis* H37Ra; TLR, toll-like receptor; ERK, extracellular-signal-regulated kinase; TNF, tumor necrosis factor; MMP, matrix metalloproteinase; Ⓟ, phosphorylate

Accordingly, the mechanism regulating MMP expression in TBP needs to be investigated. Previous studies showed that MTB infection can induce MMP-1 and MMP-9 expressions in vivo and in vitro [12, 13]. In addition, TNF-α is responsible for driving the gene expression of MMP-1 and MMP-9 in MTB-infected airway epithelial cells and monocytes, respectively [12, 13]. Our data showed that either MTBRa or TNF-α alone could stimulate protein expression of MMP-1 and MMP-9 in human PMCs, and neutralization of TNF-α substantially reduced MMP-1, but not MMP-9, elicited by MTBRa, suggesting that TNF-α is critical for MMP-1 expression in human PMCs. The previous study ever demonstrated that TNF-α is a key autocrine and paracrine regulator of MMP-9 secretion by human monocytes [12]. As our results showed that MTBRa remarkably stimulated TNF-α expression and which significantly induced MMP-1 and MMP-9 production, TNF-α may serve as an autocrine trigger for MMP secretion by human PMCs in response to MTB infection and may play a critical role in the whole pathological process of TBP (Fig 7).

However, as MTB-infected monocytes or macrophages are proposed to be the major source of TNF-α and MMP-9 in tuberculosis [12, 28], and there is emerging evidence that cell-cell interaction between immune cells and stromal cells are implicated the intercellular network to drive MMP production in tuberculosis [3]. Therefore, the current study is limited by lack of information on the relative contribution of PMCs and other immune cells to the production of TNF-α and MMPs in TBP. Further studies on the interaction between PMCs and other immune cells during the development of TBP are needed to verify this issue.

In conclusion, the present study demonstrated that MTBRa upregulates TNF-α expression through activation of TLR2/ERK signalings, and increases MMP-1 and MMP-9 production in human PMCs, which are associated with effusion volume and pleural fibrosis and may contribute to the pathogenesis of TBP. Further investigation of the manipulation of TNF-α and MMP expression in pleural mesothelium may provide new insights into the mechanisms and rational treatment strategies for TBP.
